# The Veteran Friendly Practice accreditation programme: a mixed-methods evaluation

**DOI:** 10.3399/BJGPO.2022.0012

**Published:** 2022-07-13

**Authors:** Alan Finnegan, Kate Salem, Lottie Ainsworth-Moore, Rebecca Randles, Lauren West, Robin Simpson, Veronica Benedicta Grant

**Affiliations:** 1 Westminster Centre for Research in Veterans, University of Chester, Chester, UK; 2 Hampton Surgery, Solihull, UK; 3 Darley Dale Medical Centre, Matlock, UK

**Keywords:** health promotion, mental health, general practice, primary healthcare

## Abstract

**Background:**

The Royal College of General Practitioners (RCGP) Veteran Friendly Practice Accreditation Programme launched in 2019, aiming to allow practices to better identify, treat, and refer veterans, where appropriate, to dedicated NHS services.

**Aim:**

To evaluate the effectiveness of the accreditation programme, focusing on benefits for the veteran, the practice, and the delivery of the programme itself.

**Design & setting:**

The study evaluated the views of veteran-friendly accredited GP practices across England.

**Method:**

A mixed-methods study was undertaken, which collected data via an online survey from 232 accredited primary healthcare (PHC) staff and 15 semi-structured interviews with PHC veteran leads. Interviews were analysed using modified grounded theory.

**Results:**

The study found 99% (*n* = 228) of responders would recommend the programme, 78% (*n* = 180) reported improved awareness, and 84% (*n* = 193) a better understanding of veterans' needs. Seventy-two per cent (*n* = 166) identified benefits for veterans who were engaging more with PHC but participants felt more time was needed, largely owing to the COVID-19 pandemic, to fully assess the impact of the programme on help-seeking behaviour. Challenges included identifying veterans already registered, promoting the accreditation process, and ensuring all PHC staff were kept up to date with veteran issues.

**Conclusion:**

The programme has increased signposting to veteran-specific services and resulted in greater understanding of the NHS priority referral criteria for veterans. Recording of veteran status has improved and there was evidence of a better medical record coding system in PHC practices. These findings add to the limited empirical evidence exploring veteran engagement in PHC, and demonstrate how accreditation results in better treatment and identification of veterans.

## How this fits in

Accreditation aims to better identify, treat, and refer veterans, where appropriate, to dedicated NHS services. So far, over 1000 practices have gained accreditation. This evaluation demonstrates GP practices have a greater knowledge, awareness, and understanding of veterans' needs and services as a direct result of the accreditation. The programme also led to improvements in the recording of veteran status in GP practices.

## Introduction

The NHS is responsible for the health care of an estimated 2.4 million military veterans in Great Britain,^1,2^ with the UK having witnessed considerable statutory and non-statutory investment in military veteran health services in recent years.^3^ Since 1985, the UK has utilised Read codes (which are being updated with Systematized Nomenclature of Medicine Clinical Terms [SNOMED CT] codes), which are applied to a patient’s PHC medical record to annotate demographic characteristics including age, sex, diagnosis, and therapeutic interventions.^4^ The UK’s Department of Health and Social Care asserts that a Read or SNOMED code should be applied to medical documentation indicating a *'history relating to military service'*.^5^ For the minority of veterans who need mental health (MH) support, poor help-seeking leads to unnecessary delays in addressing operationally attributable MH issues, which are often left until they are in crisis and social isolation.^6,7^ Additionally, some veterans are unaware of the potential health and social care benefits of disclosing their ex-armed forces status to their GP;^8^ only approximately 8% of veterans are registered under the correct Read or SNOMED code.^8–10^

PHC staff can positively change health behaviour patterns, but there is a lack of knowledge among them regarding veterans.^8,9^ GPs are often unaware how many veterans are registered with their practice and may require more guidance on how to meet the needs of their veteran patients.^10^ To maximise the uptake of these services, it is vital that veterans and their families register with PHC practices and are aware that these services exist.

The RCGP Veteran Friendly Practice Accreditation Programme was launched in June 2019 as part of an NHS 10-year plan to improve veteran engagement with PHC providers. The aim of accreditation was to allow practices to better identify, treat, and refer veterans, where appropriate, to dedicated NHS services, thereby demonstrating motivation and commitment to ensuring veterans and their families are provided with better patient-centred care within GP practices.^9^ It has also assisted the NHS in being able to meet the health commitments of the Armed Forces Covenant, which is the nation's pledge to acknowledge and understand the commitment and sacrifices made by veterans. It states they should be treated with fairness and respect, and not be disadvantaged in the support they receive.^11^ By October 2021, over 1000 of the 6993 English PHC practices^12^ have been accredited across England (approximately 14% of GP practices). NHS England funded the accreditation programme for an initial 3-year period with an evaluation scheduled for the final year. After an open call and then interviews of the short-listed applicants, the University of Chester’s Westminster Centre for Research in Veterans was selected to complete an independent evaluation.

### Aim and objectives

The aim of this study was to evaluate the effectiveness of the RCGP Veteran Friendly Practice Accreditation Programme to highlight positive outcomes and identify areas for improvement.

The objectives were to: a) identify GP and PHC staff assessment of the effectiveness, benefits, problems, and means for improvement of the RCGP Veteran Friendly Practice Accreditation Programme for both veterans and the practices; b) recognise the challenges of this intervention, why they exist, and how they can be positively addressed; and c) distinguish the potential for lessons learnt to improve the programme.

## Method

A survey was designed with questions aligned to the aims and objectives, and included open-ended questions to facilitate exploration of the benefits and challenges of the programme. A pilot study indicated the 25-item online survey, using the Jisc Online Surveys tool,^13^ would take approximately 10 minutes to complete. Demographic information about the practices and veteran leads was acquired, along with the impact of COVID-19. Content analysis^14^ was used to analyse the survey’s written comments, and identified the inclusion of certain words and themes to help expand on the responders’ replies. Surveys included a request for PHC staff to voluntarily take part in a short interview, which aimed to understand positive outcomes, challenges, and to determine whether the programme had improved veteran help-seeking behaviour. Qualitative data from the interviews were analysed using a modified grounded theory approach.^15–17^ This facilitated a structured and systematic means of viewing the programme from the participants’ perspectives and included the following: constructing analytical codes and categories from the data and not from preconceived suppositions; using the constant comparative method to construct comparisons during each stage of the analysis; and memo-writing to develop categories, specify their properties, define correlations, and identify gaps. The qualitative analysis complied with the EQUATOR Network resources for reporting qualitative research^18,19^ and undertaking qualitative research with a British Armed Forces population.^20^ The research team were independent of the accreditation programme. Authors 6 and 7 were the RCGP military veteran champions, and they provided direction regarding the background to the accreditation programme and helped with facilitating the data collection.

The evaluation started at the beginning of May 2021, at which time 949 practices were accredited. As some practices were multiple location practices, the number of practices recorded as accredited by the RCGP was 925. The inclusion criterion was being an RCGP-accredited practice; all were contacted. The performance indicator set by the funders was 10% of practices, which meant a minimum of 92 participants were required to complete the survey.

### Quantitative survey

Email addresses of accredited practices were held on an RCGP database, which the research team were able to access for this evaluation only. The survey link was initially sent to 50 veteran-friendly accredited practices via email to ensure they were being distributed and received correctly. All accredited practices then received the survey (*n* = 925); survey distribution is presented in Table 1.

**Table 1.** Survey distribution

Serial	Survey distribution	Date (2021)	Responses	Percentage increase %
1	Initial 50 surveys	29 April		
2	Remaining surveys	11 May	96	
3	Reminder 1	18 May	159	65
4	Reminder 2	2 June	189	19
5	RCGP newsletter	23 June		
6	Reminder 3	24 June	232	23
7	Total responses		232	

RCGP = Royal College of General Practitioners.

Survey links were individual, which allowed a unique identifier code to be assigned to each participant. Responders were able to submit questionnaires without answering all 25 questions and no incentive was offered. The timelines for the evaluation were set at approximately 2 months, so that the results were available for a review of further funding. Therefore, to increase participation — and mindful that PHC practices were under significant pressures, exacerbated by the COVID-19 pandemic — the research team with support of authors 6 and 7 arranged for automatic reminders to be sent to the email addresses of those who had not yet completed the survey (Table 1).

The RCGP included details of the survey within their quarterly newsletter, reminding accredited practices to complete the survey. Quantitative survey data were kept anonymous and confidential. Data were exported directly from Jisc Online Surveys,^13^ which was password protected and only accessible by the research team. Quantitative survey responses were analysed using IBM SPSS^21^ Statistics (version 26) and included descriptive statistics of frequency distributions and percentages with scope for cross-tabulations, allowing examination of relationships between variables.

### Qualitative interviews

Fifteen semi-structured interviews were conducted via Zoom and Microsoft Teams, and were audio-recorded using a dictaphone. This sample was based on a ‘first come, first served’ basis. Qualitative data obtained from the interviews were transcribed manually, coded, and kept anonymous and confidential.

## Results

A total of 232 practices completed the survey (25% response rate). Given the significant pressures faced by GP practices during the pandemic, this was considered an exceptional response rate. The mean total practice population was 9883 (range 1650–31 000). Some practices, however, recorded unusually high population figures. This may be owing to multiple practice locations or human error. The mean number of veterans registered at these practices was 99 (range 2–800). Again, this figure should be interpreted with caution as it may not reflect a verified SNOMED or Read code search for veteran status. Seventy-two per cent of practices (*n* = 167) recorded the number of veterans in their practice but only 18% (*n* = 42) included a veteran in their patient participation group. Of 230 responses, 95% (*n* = 219) of practices had a veterans lead, of whom 33% (*n* = 75) were veterans themselves. Of the 219 veteran leads, the most common appointment held was GP 69% (*n* = 151), followed by nurse 13% (*n* = 29), and practice manager 7% (*n* = 15).

Ninety-nine per cent (*n* = 228) of participants would recommend the accreditation, with 76% (*n* = 176) finding the accreditation process ‘easy’ or ‘very easy’. Communication with the RCGP during the accreditation process was perceived as ‘good’ or ‘very good’ by 74% (*n* = 170), and an online module was the preferred method of training material for 84% (*n* = 194). Since becoming accredited, 84% (*n* = 193) of responders had a greater understanding of veterans' needs, while 78% (*n* = 180) were more aware of veteran’s needs. Only 52% (*n* = 120) of participants believed veterans were ‘aware’, ‘very aware’, or ‘somewhat aware’ of the programme, while 67% (*n* = 154) felt veterans did have an awareness of the priority treatment system. Seventy-two per cent (*n* = 166) of survey responders believed their increased understanding was having a positive impact on veterans. Sixty-eight per cent (*n* = 123) of overall responses suggested the accreditation process was the most effective way of providing veteran leads with the experience they needed to support veterans. Quantitative survey data are presented in Table 2, followed by integrated findings from both quantitative and qualitative data analysis in Table 3.

**Table 2.** Descriptive statistics of quantitative results

Serial	Theme	Question	Total responses, *n*	‘Yes’ answers, *n*	%
**1**	**Impact on practice**	**Greater understanding of veterans**	229	193	84
**2**		**More aware of veterans' needs**Very awareAwareSomewhat awareSlightly awareNot at all aware	232	6211838122	27511651
**3**		**Impact of programme on practice** (*0 no impact–10 significant impact*)	231 (mode 5; mean; 5; median 5; standard deviation 2.53)		
**4**	**Impact on veteran**	**Age group most likely to engage**18–3940–5960–79≥80All equally likely to engage	230	248943668	103919330
**5**		**Veteran awareness of programme**Very awareAwareSomewhat awareSlightly awareNot at all aware	231	638767140	317333117
**6**		Veteran awareness of veteran-specific priority treatmentVery awareAwareNeutralSome awarenessUnaware	231	675597318	33326328
**7**		**Perceived benefit to veteran of their GP having greater understanding of needs**Significant benefitSome benefitNeutralLittle benefitNo benefit	231	4412246145	19532062
**8**		**Impact of programme on veteran** (*0 no impact–10 significant impact*)	232 (mode 5; mean 5.60; median 6; standard deviation 2.35)		
**9**	**Programme management**	**Would recommend accreditation programme**	231	228	99
**10**		**Accreditation process**Very easyEasyNeutralDifficultVery difficult	232	601165330	26502310
**11**		**Communication with RCGP during accreditation process**Very goodGoodNeutralPoorVery poor	230	431275451	19552420.4
		**Preferred training material** (*multiple response question*)Online moduleFace-to-face courseRCGP veteran-specific newsletterWebsite updatesOther (central resource database or contacts with veteran organisations)		194631801396	842777603

RCGP = Royal College of General Practitioners.

**Table 3.** Integrated findings from both quantitative and qualitative data

Theme	Quantitative results	Qualitative findings	Integration
Programme delivery and implementation	Well received by PHC staffEasy to become accreditedUseful information from RCGP, preference for online educational modules	No qualitative data	N/A, quantitative data provided insight into this area
Impact on veteran	PHC staff believed veterans aged 40–59 years most likely to engagePHC staff believed veterans were largely unaware of the programme and of veteran-specific priority treatment	Greater understanding of veterans needsGreater appreciation of veteran-specific servicesIncreased engagement with PHCSome improvement in help-seeking	Important for PHC staff to have an understanding of veteran-specific services to bridge the gap between patient knowledge and services availableQualitative findings expanded on quantitative data about age and how this might affect help-seeking
Impact on practice	Greater appreciation and awareness of veterans' needs	Increased veteran registrationsGreater efforts to code correctlyBetter working environment	Becoming aware of veterans' needs has had a positive impact on motivation and commitment to identify veterans
Challenges	COVID-19Identifying veteransPoor secondary servicesKeeping up to date	Identifying veteransPromoting accreditation statusFurther training needs	COVID-19 has impacted footfall, subsequently affecting communicating accreditation status to veteranFurther work required outside of PHC to complement veteran-friendly practicesFuture training should be aimed at all PHC staff, not just veteran leads
Positive outcomes	No quantitative data	Increased awareness of veterans' needsGreater understanding of veteran-specific servicesImproved understanding of the priority referral system	N/A, qualitative data provided insight into this area

N/A = not applicable. PHC = primary health care.

### COVID-19 impact

The survey included an open-ended question about the impact of COVID-19 on the programme. Of 71 responses, 48% (*n* = 34) felt the greatest challenge presented by the pandemic was limited time, 24% (*n* = 17) reported the lack of footfall, while 15% (*n* = 11) felt the reduced communication with patients made implementing the programme more difficult.

### Qualitative findings

**Figure 1.**
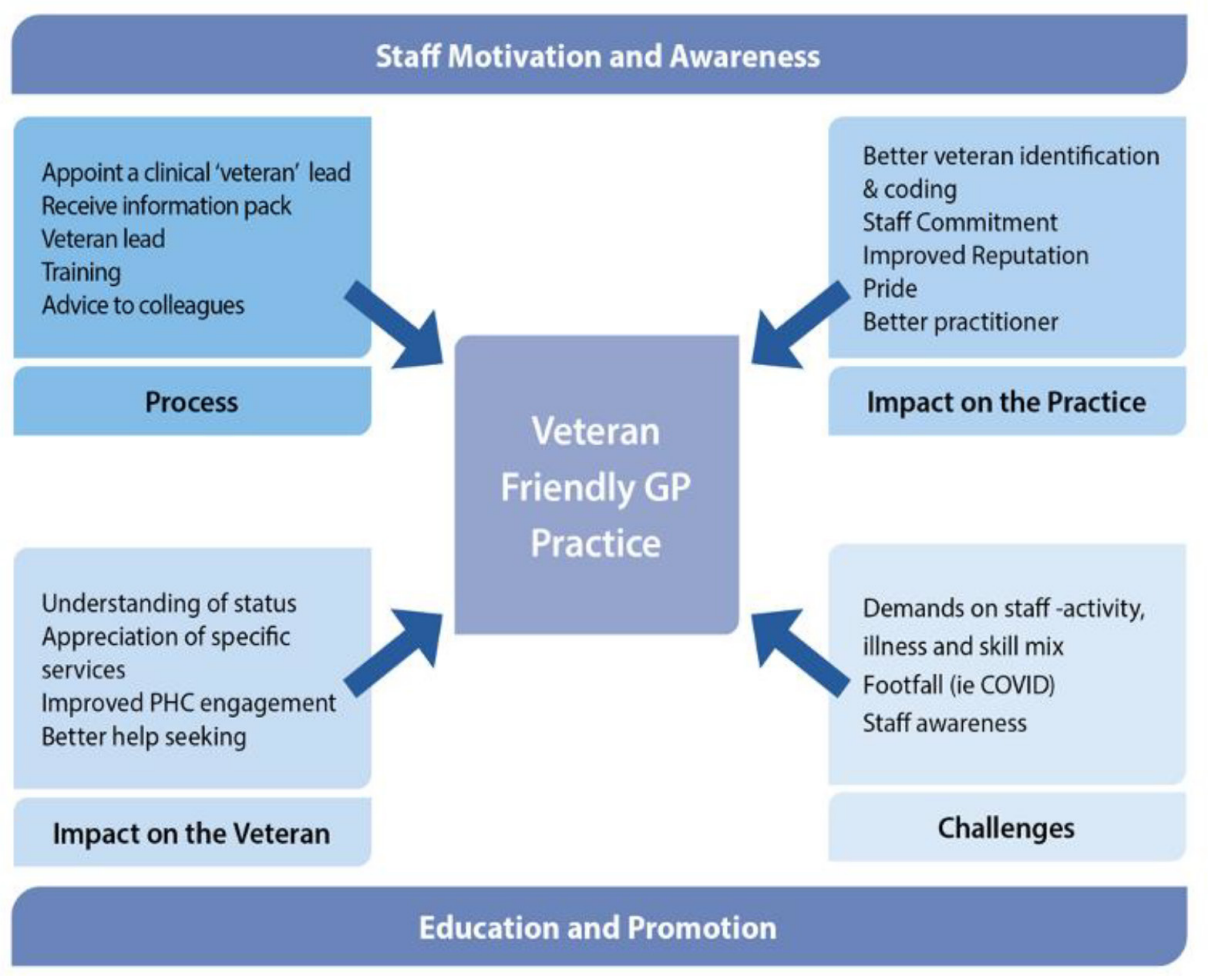
Factors influencing accreditation and outcomes of the programme. PHC = primary health care

Analysis identified factors influencing the process, impact on the veteran, impact on the practice, and challenges (shown in Figure 1). Findings from the open-ended survey questions and qualitative interviews give greater meaning to the quantitative survey results. Using different data from accredited-PHC practices enabled more in-depth interpretation of the data and strengthens conclusions about the programme. Presentation of the findings is designed to protect the anonymity of responders by coding their responses (for example, AA, BB) and no further information is provided.

### Impact on the veteran

#### Understanding veterans’ needs

Accreditation was perceived to have a positive impact on military veterans, with 67% (*n* = 10) of interviewees reporting a greater appreciation of veterans' needs since becoming accredited; 72% (*n* = 166) of survey responders believed this increased understanding benefitted veterans. GP practices became more understanding of veterans' experiences and were more aware of their need for veteran-specific care:

*'Given the fact that he was a veteran with mental health issues, I did take time to explore his needs and what we can offer him. He was somewhat taken a back, I think it wasn’t something he expected. I explored his needs, his mental health, his friendships, his need for a system as a veteran.'* (BB)

This resulted in more successful engagement with secondary health care. Similarly, interviewees believed their increased understanding had led to an improved doctor—patient relationship. Improved understanding was also demonstrated by nursing and administrative staff.

#### Understanding veteran-specific services

Ten interviewees had a better understanding of veteran-specific services since becoming accredited, particularly in relation to MH and orthopaedic referrals:

*'Just having an awareness of what services are available, I didn’t know about things they could access — particularly with mental health. Now I know where I can refer that veteran who really needs that help.'* (FF)

This knowledge was informed by RCGP updates and self-motivated online learning (webinars and veteran-specific websites). Overall, accredited practices have a good appreciation of the Armed Forces Covenant priority referral process and utilise this well. They felt it was being implemented by secondary health care, although there were isolated instances requesting further education to inform both primary and secondary care services about priority treatment:

*'I didn’t understand what priority treatment is and I don’t think NHS trusts do, so they don’t follow it through on a secondary level. So, if I put a referral through and he’s in chronic pain, I don’t think they’d register it more than if it was a truck driver or a builder, to them they’re all the same.'* (LL)

Eleven interviewees believed veterans were unaware of the veteran-specific services available, and GPs have essentially ‘*bridged*’ the gap between the veteran and available services, utilising veteran-specific services that would not otherwise have been accessed by the veteran:

*'It’s me knowing it exists rather than patients coming to me telling me it exists … if I wasn’t there, I guess they would just get referred in the standard NHS way and longer waiting times and a less bespoke service.'* (EE)

Several interviewees had developed a veteran resource database in their practice since becoming accredited, utilising it as a foundation to access guidance regarding signposting their veteran patients to the most appropriate service.

#### Veteran help-seeking and engagement

Five interviewees reported a small but definite increase in veteran help-seeking since becoming accredited, especially in younger veterans:

*'I think it has made veterans more likely to come forward and seek help … I think a lot of patients are the type of patients who wouldn’t normally come to seek help. They tend to be younger to middle-aged men really, which you don’t see a lot of.'* (JJ)

Participants felt it was easier to communicate with older veterans once they became aware their GP had an understanding of their military experience. Older veterans were considered to have little appreciation of their veteran status, with some being unaware they were classed as a veteran. Survey data supports this finding, whereby only 3% (*n* = 6) of survey responders believed veterans aged >80 years would engage with an accredited practice. This reinforces previous findings highlighting a need for further exploration into the needs of older military veterans.^22^ Eight interviewees reported that veterans had registered with practices directly as a result of hearing about their accreditation:

*'We have patients who have registered with our practice deliberately because we are veteran friendly or transferred to our practice from other practices for that reason.'* (CC)

As a direct result of the initiative, GP practices were better able to understand veterans' needs and have a greater awareness of how to meet them. Overall, GP practices have a good appreciation of the Armed Forces Covenant priority referral process and utilise this well. Veterans themselves are perceived to lack understanding of veteran-specific services, which highlights the importance of the GP practice’s veteran-specific knowledge and consideration. However, it is important to note that in the qualitative aspect of the evaluation, 10 interviewees were veterans themselves, which makes it difficult to assess whether their knowledge was influenced by their own military experiences. Indeed, interviewees who were veterans appeared to have greater access to priority health care as they had forged military connections within other health and social care services. Nevertheless, interviewees who were not veterans were equally motivated to use both priority referrals and veteran-specific services in order to maximise the care and support to their veteran clients.

Seven interviewees believed it was too early to gauge the impact of the accreditation on veteran help-seeking, largely owing to COVID-19, and felt this evaluation may be better timed in another 12 months. This was also evidenced in the open-ended survey responses.

### Impact on the practice

#### Veteran registrations

Since becoming accredited, some GP practices reported an increase in veteran registrations:

*'We’ve certainly seen more veterans. People have deliberately registered with us as a result of the accreditation including some who have been out of area who we wouldn’t normally register. They’re wanting to see somebody who understood the military and could assist them more.'* (NN)

However, participants believed there was work still to be done to identify veteran patients, believing they needed more time to increase their veteran registrations. This was in part pre-empted by COVID-19. Nevertheless, participants felt that improved awareness had resulted in practice staff who were more motivated to identify veterans and willing to accept and adopt new strategies. Reception staff asked new patients, *'Have you ever served*?', while nursing and GP staff asked patients during consultations whether they were veterans, and this was positively received by veteran patients. This form of proactive engagement was not done before accreditation. Awareness of veteran patients subsequently led to improved coding of veterans:

*'I’ve been a lot more aware of registering veterans as the right Read Code … I’ve registered a lot more as the right Read Code since becoming accredited.'* (FF)

Motivation to register veterans appeared to be guided by a desire to improve veteran health care, although two interviewees reported that their clinical commissioning group had not been allocated funding to undertake veteran-specific coding searches, suggesting that funding may be a motivator to further improve PHC engagement.

#### Working environment

Eleven interviewees described how PHC staff’s motivation and commitment to the programme invoked a sense of pride in all practice employees. This was considered to have benefitted the reputation of the practice:

*'When we first got registered, there was huge support from the staff, particularly the admin team. They were really positive, quite a few of them have got spouses who are ex-military or children in the military. It raised the profile of military awareness. We have a real sense of pride in the staff, supporting the military.'* (CC)

Several interviewees felt they had become a better practitioner as a result of the understanding and awareness gained from being accredited. Reinforcing survey results, all practice staff were described as having an improved appreciation of veterans' needs:

*'The main thing is having somebody in the surgery who understands them. It’s not just me, it’s the fact the receptionists are more understanding … It’s allowed us to be more focused and because of the fact they know about the different referral pathways, we can often refer them into a service where they get a better answer, especially for mental health.'* (NN)

The accreditation itself has had a positive and beneficial impact on GP practices. Participants felt accreditation resulted in increased veteran registrations, improved SNOMED and Read coding systems, and greater motivation to identify veterans. While there remains work to be done in terms of reaching all patients, the fact that improvements have been observed should be considered a positive outcome. A better working environment was also noted and staff motivation to improve veteran health care highlighted. This is an encouraging finding and suggests continuation of this programme would be warmly received by PHC staff.

### Challenges

#### Identifying veterans

Identifying veterans so they could be correctly coded on their medical records and made aware of the accreditation status was a primary concern for seven interviewees and was also highlighted consistently in the survey data:

*'We are quite new to this so numbers are fairly small but we found several veterans who were relatively new out of service and we’ve been able to engage with them and they’ve been very positive about it, it’s been quite helpful for them.'* (II)

Reaching these veterans was more problematic owing to the lack of footfall as a result of COVID-19. Several participants also raised concerns about veterans’ understanding of their status. Interviewees suggested many veterans had little appreciation of the benefits associated with disclosing their status to their GP, highlighting a need to promote understanding of veteran status in the veteran community.

#### Promoting veteran-friendly accreditation

PHC practices advertised their accreditation on their website, introduced veteran-specific material to posters and to the public health information shared on their internal TV screens in their practice waiting rooms and through local communication by 'word of mouth'. However, 11 interviewees felt these methods of advertising were not having maximum impact and were considering alternative ways of promoting their accreditation status. Interviewees believed ‘word of mouth’ was the most effective method of promoting their accreditation but acknowledged this would take time:

*'I’m always looking for ways to help veterans. We are quite proactive. Veterans aren’t even aware that practices are veteran friendly so they’ve been quite shocked. I’ve put messages out to the Armed Forces Community that I’m in touch with, the armed forces breakfast clubs and things. A couple of our patients, when they’ve moved into area and gone along to armed forces breakfast clubs, they’ve said the surgery is veteran friendly so we’ve had a few patients come down and register.'* (GG)

#### Training needs

Ensuring all practice staff were made aware of the programme was considered an additional responsibility for veteran leads, especially for practices with a high turnover of staff. Six interviewees advocated a need for further training, and the survey results revealed GP practices found RCGP updates extremely useful, although there were recommendations for these to be received on a more regular basis. This might suggest a need for more frequent veteran-specific updates from the RCGP and the NHS. Others suggested further training would be beneficial such as a conference, meetings, and/or study days.

GP practices have found it difficult to reach veterans as a result of COVID-19 and have had little time to implement changes, although the veterans they have reached have benefitted greatly from the programme. In addition, GP practices may profit from further opportunities to enhance their knowledge of how best to meet the needs of veterans and ensure they receive the optimum level of care.

## Discussion

### Summary

Since 2019, over 1000 GP practices have gained veteran-friendly accreditation status. The positive outcomes of this independent evaluation have demonstrated the importance of the accreditation programme. Veterans receive better PHC when PHC staff understand their needs, receiving improved signposting to veteran-specific statutory and non-statutory services. Veteran leads’ enhanced knowledge of veteran-specific services and the priority referral pathway is of great benefit to veterans, who themselves have little understanding of these provisions.

Recording of veteran status in GP practices has improved and staff appear motivated to engage with the programme. As time goes on, the number of veterans registering with accredited practices will likely increase. However, reaching veterans already registered (but not correctly coded) and promoting the accreditation remains a concern. Some GP practices have developed veteran-specific resource databases, highlighting commitment from PHC staff to engage with the programme. A better understanding of the coding system is evident in GP practices, although correct coding of veterans is an ongoing process. GP practices felt they needed more time to fully assess the programme’s impact on veteran help-seeking behaviour.

### Strengths and limitations

This evaluation adds to the limited empirical evidence exploring the effectiveness of veterans’ engagement in PHC and staff’s willingness to connect with veterans. Recording of veteran status in GP practices has improved and momentum to do so is likely to continue. PHC veteran leads’ knowledge of veteran-specific services and the priority referral pathway is beneficial to veterans, who themselves have little understanding of these provisions. The study presents an exceptional response rate from GP practices who were experiencing great pressure as a result of the COVID-19 pandemic.

The recruitment approach taken for the qualitative interviews meant the PHC practices that answered the questionnaire had both applied for accreditation and so were motivated to complete the survey questionnaire, which may have influenced the results. In addition, 10 of the 15 interviewees were military veterans themselves, meaning that they may have had a greater understanding of veterans' experiences than non-veteran interviewees. The effect of the COVID-19 pandemic on PHC will have impacted on the results. This could have been improved by identifying the number of veterans registered before accreditation and the number registered at a fixed time period (for example, 6 months) after accreditation.

### Comparison with existing literature

There is a lack of international literature exploring veteran engagement with PHC, hence the RCGP funding this evaluation. Within the UK, patients are seldom asked whether they have ever served in the armed forces and recording of veteran status was, before this programme, rarely documented or coded,^8–10^ which makes identifying this population particularly difficult. Part of the challenge is that veterans are a hard-to-reach group^23^ who often bottle up their feelings, fearing the impact of sharing personal burdens with their family or appearing weak.^24^ Veterans may believe that civilian health professionals will not understand their past military experiences and therefore not register with a PHC practice, or not disclose their veteran status.^25^

### Implications for research and practice

This evaluation adds to the limited empirical evidence exploring the effectiveness of veterans’ engagement in PHC and staff’s willingness to connect with veterans. It highlights better treatment and identification of veterans since becoming accredited, while commitment from practice staff demonstrates the possibility of further developing the Veteran Friendly Practice Accreditation Programme. These factors have important implications for future policy development, research, educational programmes, and clinical delivery. The combination of these will help ensure veterans and their families receive the optimum care and support that they deserve. Study recommendations are shown in Table 4.

**Table 4.** Study recommendations

Recommendations
1. Educational provision. To evaluate the current online educational packages and update and improve accordingly. Educational material should target all practice staff.
2. Specific development opportunities for veteran leads. Examples include developing networks across the primary care networks and connecting the veterans leads to their regional Armed Forces Covenant partnership committee.
3. More time to fully assess the impact of the programme. As highlighted in the limitations section, the timing of the study during the COVID-19 lockdown period and the reduced footfall within PHC will have impacted on the results.
4. Identify ways in which to better promote veteran-friendly accreditation.
5. Raise awareness of veteran status in the veteran community.
6. Research. Further studies could target help-seeking and engagement from certain demographic groups such as sex, age, minority groups, and families.
7. There is clear evidence regarding the benefits of the programme to warrant continuation of the project and further funding.

PHC = primary health care.

## Data

All data on which the conclusions of this research rely are presented in the article.

## References

[1] Ministry of Defence (2017). Annual population survey: UK Armed Forces veterans residing in Great Britain 2017. https://www.gov.uk/government/statistics/annual-population-survey-uk-armed-forces-veterans-residing-in-great-britain-2017.

[2] NHS.UK (2019). Healthcare for the armed forces community. https://www.nhs.uk/nhs-services/armed-forces-community.

[3] Bacon A, Martin E, Swarbrick R, Treadgold A (2022). National health service interventions in England to improve care to armed forces veterans. BMJ Mil Health.

[4] NHS Digital (2017). Read Codes. https://digital.nhs.uk/services/terminology-and-classifications/read-codes.

[5] Royal College of General Practitioners, Royal British Legion, Combat Stress (2011). Meeting the healthcare needs of veterans. A guide for general practitioners. https://www.chester.ac.uk/sites/files/chester/MeetingTheHealthcareNeedsOfVeteransLeaflet.pdf.

[6] Combat Stress (2016). Annual report and accounts. https://combatstress.org.uk/about-us/annual-report-and-accounts.

[7] Randles R, Finnegan AP (2022). Veteran help-seeking behaviour for mental health issues: a systematic review. BMJ Mil Health.

[8] Finnegan AP, Jackson R, Simpson R (2018). Finding the forgotten: motivating military veterans to register with a primary healthcare practice. Mil Med.

[9] Simpson RG, Leach J (2015). The general practitioner and the military veteran. J R Army Med Corps.

[10] Finnegan A, Randles R (2022). Prevalence of common mental health disorders in military veterans: using primary healthcare data. BMJ Mil Health.

[11] Ministry of Defence (2020). Armed Forces Covenant annual report. https://www.gov.uk/government/publications/armed-forces-covenant-annual-report-2020.

[12] Bostock N (2019). Number of GP practices in England falls below 7000. https://www.gponline.com/number-gp-practices-england-falls-below-7000/article/1525443.

[13] Jisc (2021). Online surveys. https://www.onlinesurveys.ac.uk.

[14] Burnard P (1991). A method of analysing interview transcripts in qualitative research. Nurse Educ Today.

[15] Charmaz K (2014). Constructing Grounded Theory.

[16] Finnegan A, Finnegan S, Thomas M, Deahl M (2014). The presentation of depression in the British Army. Nurse Educ Today.

[17] Glaser BG, Strauss AL (1999). The discovery of grounded theory: strategies for qualitative research.

[18] Equator Network (2022). Enhancing the QUAlity and Transparency Of health Research. https://www.equator-network.org/reporting-guidelines/srqr/.

[19] O’Brien BC, Harris IB, Beckman TJ (2014). Standards for reporting qualitative research: a synthesis of recommendations. Acad Med.

[20] Finnegan AP (2014). Conducting qualitative research in the British Armed Forces: theoretical, analytical and ethical implications. J R Army Med Corps.

[21] IBM (2021). IBM SPSS statistics. https://www.ibm.com/uk-en/products/spss-statistics.

[22] Di Lemma LCG, Finnegan A, Howe S (2022). Critical analysis of the Armed Forces Covenant Fund Trust Aged Veterans Fund. BMJ Mil Health.

[23] Drabble D, Allen R, Child C (2019). The Armed Forces Community Navigation Project. https://s31949.pcdn.co/wp-content/uploads/armed-forces-community-healthcare-navigation-project-feasibility-study.pdf.

[24] Ahern J, Worthen M, Masters J (2015). The challenges of Afghanistan and Iraq veterans’ transition from military to civilian life and approaches to reconnection. PLoS One.

[25] Improving Access to Psychological Therapies (IAPT) (2013). Veterans: positive practice guide. https://kirkleesiapt.co.uk/wp-content/uploads/2018/05/veterans-positive-practice-guide-2013.pdf.

